# Large-Scale Bi-Level Strain Design Approaches and Mixed-Integer Programming Solution Techniques

**DOI:** 10.1371/journal.pone.0024162

**Published:** 2011-09-09

**Authors:** Joonhoon Kim, Jennifer L. Reed, Christos T. Maravelias

**Affiliations:** 1 Department of Chemical and Biological Engineering, University of Wisconsin-Madison, Madison, Wisconsin, United States of America; 2 DOE Great Lakes Bioenergy Research Center, University of Wisconsin-Madison, Madison, Wisconsin, United States of America; The Centre for Research and Technology, Hellas, Greece

## Abstract

The use of computational models in metabolic engineering has been increasing as more genome-scale metabolic models and computational approaches become available. Various computational approaches have been developed to predict how genetic perturbations affect metabolic behavior at a systems level, and have been successfully used to engineer microbial strains with improved primary or secondary metabolite production. However, identification of metabolic engineering strategies involving a large number of perturbations is currently limited by computational resources due to the size of genome-scale models and the combinatorial nature of the problem. In this study, we present (i) two new bi-level strain design approaches using mixed-integer programming (MIP), and (ii) general solution techniques that improve the performance of MIP-based bi-level approaches. The first approach (SimOptStrain) simultaneously considers gene deletion and non-native reaction addition, while the second approach (BiMOMA) uses minimization of metabolic adjustment to predict knockout behavior in a MIP-based bi-level problem for the first time. Our general MIP solution techniques significantly reduced the CPU times needed to find optimal strategies when applied to an existing strain design approach (OptORF) (e.g., from ∼10 days to ∼5 minutes for metabolic engineering strategies with 4 gene deletions), and identified strategies for producing compounds where previous studies could not (e.g., malate and serine). Additionally, we found novel strategies using SimOptStrain with higher predicted production levels (for succinate and glycerol) than could have been found using an existing approach that considers network additions and deletions in sequential steps rather than simultaneously. Finally, using BiMOMA we found novel strategies involving large numbers of modifications (for pyruvate and glutamate), which sequential search and genetic algorithms were unable to find. The approaches and solution techniques developed here will facilitate the strain design process and extend the scope of its application to metabolic engineering.

## Introduction

Metabolic engineering of microbial strains has been of great interest for producing a wide variety of chemicals including biofuels, polymer precursors, and drugs. While conventional metabolic engineering approaches often focus on modifications to the desired and neighboring pathways, recent developments in computational analysis of metabolic models allow identification of genetic modifications needed to improve production of biochemicals [1], [2], [3]. Computational approaches, such as BNICE [4] and BioPathway Predictor [5], have been developed which enumerate novel biochemical routes for chemical production. Metabolic pathway-based approaches, such as elementary modes [6] and extreme pathways [7], have been used to design strains with improved chemical production (e.g., ethanol and carotenoids [8], [9]). Subsequent analysis of elementary modes finds those with desired behaviors and finds the genetic strategies that would force cells to utilize these desired modes [10]. While advances have improved the efficiency of these pathway-based approaches, enumerating these pathways for genome-scale metabolic networks is still a very challenging task [11].

To avoid this computational challenge, approaches like flux balance analysis (FBA) [12], minimization of metabolic adjustment (MOMA) [13], and regulatory on/off minimization (ROOM) [14] use optimization to predict knockout mutant phenotypes. For example, MOMA was used to find knockout mutations that would improve lycopene and valine production in *Escherichia coli* [15], [16]. In these studies, either an exhaustive search (all possible combinations are evaluated) or sequential search (where a strategy with k+1 deletions is identified by evaluating the best strategy with k deletions combined with all single deletions) was used to find mutants with the highest predicted production using MOMA. A more recent bi-level approach based on MOMA was developed (OptGene) [17], which uses a genetic algorithm to find mutants with improved production, and this approach was used to improve sesquiterpene production in *Saccharomyces cerevisiae* [18].

A number of bi-level strain design approaches use mixed-integer programming (MIP) to efficiently identify the mutations needed to achieve the highest production rates, including OptKnock, OptStrain, OptReg, OptForce, and OptORF. These bi-level MIP approaches consist of an ‘outer’ problem and an ‘inner’ problem, where the outer problem optimizes an engineering objective function and the inner problem optimizes a cellular objective function. Frequently, the inner problem is FBA which is a linear programming (LP) problem. In MIP-based approaches, the inner FBA problem is converted into optimality constraints by formulating a dual LP of FBA and enforcing strong duality [19]. OptKnock [19] identifies a set of reaction deletions which couple cellular growth and biochemical production, so an increase in mutant growth rate requires an increase in biochemical production as predicted by FBA. Due to this coupling, adaptive evolution of these strains, where higher growth rates are selected, leads to higher biochemical production [20]. Another approach, OptStrain [21], uses a multi-step process to first identify non-native reactions that would improve the host organism's maximum production capabilities. Reaction deletions can then be found which couple production and growth in the modified host metabolic network. In addition to reaction deletions, OptReg [22] identifies reaction activations or inhibitions (increase or decrease in fluxes) to suggest up-regulation or down-regulation of metabolic genes for enhancing biochemical production. Recently, this MIP problem was reformulated and solved efficiently using a successive linear programming approach (EMILiO) [23]. Another MIP-based bi-level approach, OptForce [24], searches for all possible reaction modulations to meet a pre-specified overproduction target and identifies a minimal set of flux changes that need to be forced through genetic manipulations. Recently, we developed an approach, OptORF [25], that identifies gene deletion and overexpression strategies (instead of reaction deletions) by directly taking into account gene to protein to reaction association and transcriptional regulation. All of these bi-level MIP approaches, except OptForce, predict mutant behaviors by finding solutions that maximize growth, and so resulting strains would often need to undergo adaptive evolution to improve growth and chemical production, but they may have increased stability [20]. On the other hand, strains that have been designed using MOMA would not require adaptive evolution and for some compounds non-evolutionary strategies may be needed if product and biomass formation cannot be coupled. So the choice of computational approach will likely depend on the product of interest and experimental strategies used for strain development.

**Figure 1:**
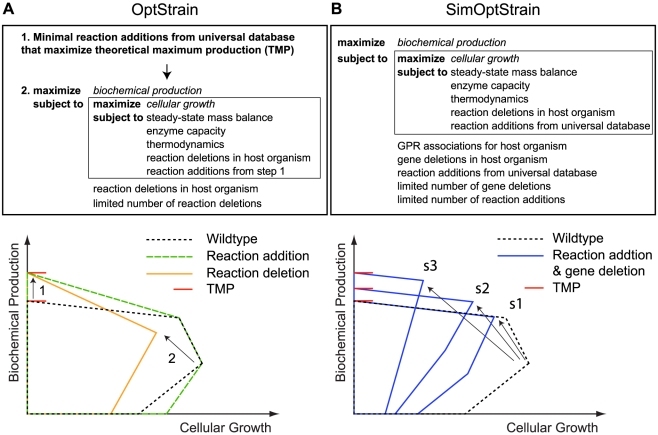
Bi-level approaches considering additions and deletions. (A) Simplified representation of the existing OptStrain procedure and an illustrative example. Step 1 adds a minimum number of reactions from a universal database that yields the maximal increase in theoretical maximum production (TMP). Step 2 identifies reaction deletions in the augmented network identified in Step 1 that couple biomass and biochemical production. (B) SimOptStrain with simultaneous gene deletion and non-native reaction addition, and illustrative examples. Solution s1 shows an example of reaction additions, which do not increase the TMP, that improve biochemical production at the maximum growth rate when combined with gene deletions. Solution s2 is an example of reaction additions that yield a suboptimal increase in the TMP, while solution s3 is a case where the number of added reactions is not necessarily the minimum. Solutions s1, s2, and s3 could only be found using SimOptStrain.

In this study, we report two new MIP-based bi-level strain design approaches and solution techniques to improve their runtime performance. First, we present SimOptStrain which simultaneously considers gene deletions in a host organism and reaction additions from a universal database such as KEGG [26] or MetaCyc [27]. Previously, the OptStrain framework used a multi-step procedure to first identify a minimal set of non-native reactions to add to the metabolic network to achieve the theoretical maximum production (TMP) of a biochemical target (Step 1 in Figure 1A), and then identify deletion strategies in the expanded metabolic network using OptKnock (Step 2 in Figure 1A). The current multi-step OptStrain procedure may miss higher production strategies by not evaluating additions and deletions simultaneously. First, additions of non-native reactions that yield zero (Solution s1 in Figure 1B) or suboptimal (Solution s2 in Figure 1B) increases in the TMP of a host organism are not considered, even though such reactions may increase the biochemical production when coupled to cellular growth in a mutant strain. Second, addition of the minimal number of non-native reactions may not lead to the highest chemical production that can be found when coupled to cellular growth rate (Solution s3 in Figure 1B). To overcome these limitations, we developed a new bi-level MIP approach which simultaneously identifies gene deletions in a host organism and reaction additions from a curated universal database, and demonstrate the utility of the approach for production of succinate and glycerol.

**Figure 2:**
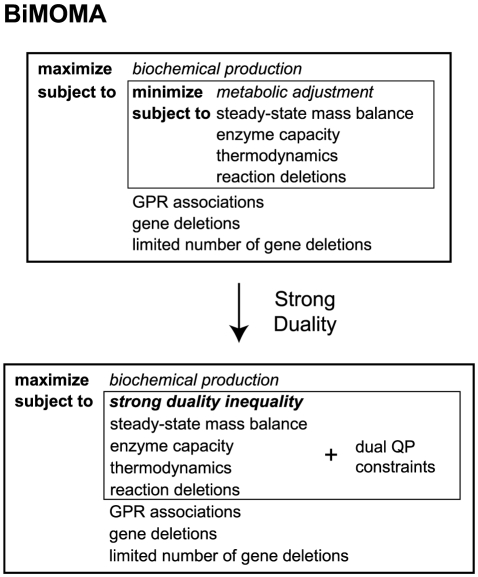
BiMOMA – a direct mixed-integer programming approach for quadratic bi-level strain design. The MOMA inner problem, a convex quadratic program, is converted to its optimality conditions using strong duality. The resulting BiMOMA problem is a single level mixed-integer quadratically constrained program.

Second, we present a new quadratic bi-level MIP approach, BiMOMA, to identify gene deletions for improving biochemical production when MOMA is used as an inner problem (see Figure 2). MOMA has been used in metabolic engineering for predicting metabolic flux distributions in un-evolved deletion mutants, and resulting strains do not need to undergo adaptive evolution. Previous studies [15], [16], [18] employed a sequential search or heuristic algorithms, such as genetic algorithms (used by OptGene), to identify gene knockout mutants with improved biochemical production. However, these approaches can be computationally expensive and may miss higher production strategies since the number of possible combinations is extremely large and the optimality of such methods is generally not guaranteed. Here, we develop a direct bi-level approach using mixed-integer quadratically constrained programming (MIQCP) and show we can efficiently identify knockout strategies for improved production of glutamate and pyruvate.

These bi-level computational approaches lead to MIP formulations that become intractable when the number of allowed modifications is large. Pre-processing and heuristic algorithms have been used to improve tractability [17], [28]; however, these methods sometimes converge to local optima and can miss better solutions. Here, we show how novel MIP techniques based on duality can significantly improve the performance of strain design approaches. We first illustrate the improvement in performance by applying the developed techniques to OptORF and comparing the results to those obtained using heuristic algorithms. We then apply the MIP techniques to the two new bi-level strain design approaches. In this work, we use ‘approaches’ to describe the strain design problems and ‘techniques’ to refer to the MIP solution methods used to solve the bi-level problems.

## Materials and Methods

### Illustration of Proposed Mixed-integer Programming Techniques using OptORF

We recently developed a bi-level optimization approach (OptORF) which uses metabolic and transcriptional regulatory models to find metabolic and/or regulatory gene perturbation strategies [25]. Using OptORF without regulatory considerations (see Text S1 for complete formulation), we demonstrate in this work how our MIP techniques can be used to quickly find global or near-global optimal solutions. The modified OptORF problem searches for metabolic gene deletion strategies to improve biochemical production, where the inner problem is an FBA problem maximizing cellular growth. The MIP techniques are described below and include four steps: tightening dual variable bounds, adding perturbation penalties, reducing search space, and solving successive problems.

#### Tightening the bounds on dual variables

First, we tightened the bounds on a subset of variables in the dual LP of FBA by examining its feasible region. Similar to FBA, the dual LP often has alternate optimal solutions due to the redundancy in metabolic networks. In a bi-level problem, any optimal solution of the dual LP will provide a feasible solution to the bi-level problem without affecting solutions of the primal LP since the primal-dual LP pair is only connected via strong duality. Therefore, we can obtain a valid solution of the dual LP among alternate optimal solutions by minimizing the norm of the dual variables subject to the dual LP constraints and optimal objective function value. We focused on dual variables corresponding to the reaction removals, and sampled their values using 1,000,000 samples of 10 random gene knockouts. We initially tested different sample sizes and numbers of gene knockouts and found the results were consistent above ∼100,000 samples and ∼5 gene knockouts. Therefore, we collected 1,000,000 samples, which was computationally tractable, and 10 gene knockouts, which was the maximum number allowed for the case studies in this work.

For each sample, we randomly choose 10 genes and solve FBA where the reactions corresponding to the 10 genes are removed via gene to protein to reaction (GPR) associations. If the FBA problem is feasible and biomass production is positive, we then minimize the Euclidean norm of the dual variables for reaction removals in the dual LP while the objective function is constrained to be equal to the optimal biomass production value. This process is repeated 1,000,000 times to sample the values of dual variables for removed reactions in different modified network structures.

**Figure 3:**
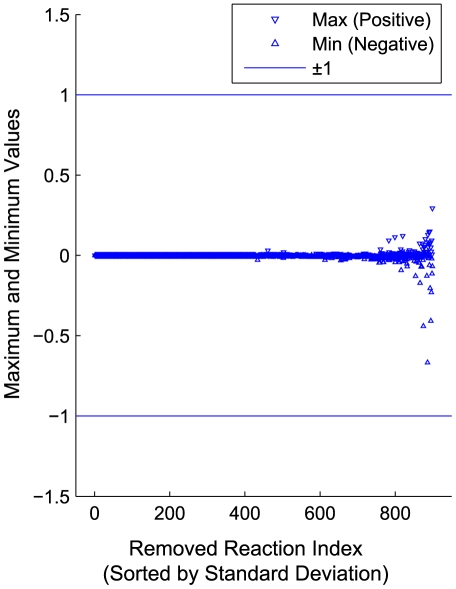
Analysis of dual variables for reaction removals using dual LP of FBA. Maximum (downward triangle) and minimum (upward triangle) of observed dual variable values for each reaction sorted by the standard deviation. The values of dual variables were obtained from 1,000,000 samples of 10 gene knockouts in glucose anaerobic condition using the *i*AF1260 metabolic model of *E. coli*.

Figure 3 shows the minimum and maximum of dual variable values (y-axis) for each reaction (x-axis) across the 1,000,000 samples of 10 gene knockouts in glucose anaerobic conditions using the *i*AF1260 metabolic model of *E. coli* [29] (see Figure S1). It can be seen from the Figure 3 that the dual variable values for non-essential reactions would not likely exceed values of +/−1. Based on these results, we tightened the bounds on these dual variables for reaction removal constraints to be [−1, 1] (Equations A.4 and A.15 in Text S1). This procedure took a few hours for 1,000,000 samples, but it only needs to be performed once for a given metabolic network and environmental conditions, and the results can be used for production of any biochemical. In this study, we did not find any cases where these bounds affected the optimal solutions of the inner FBA problem.

#### Applying penalties for genetic perturbations

Second, we applied a penalty (α) for each additional gene deletion in the outer objective function to create a trade-off between biochemical production and the required number of genetic modifications (Equation A.1). This penalty results in selection of strategies with fewer modifications among solutions with equal production and reduces the solution time.

#### Reducing the search space

Third, as other studies have done [28], [30], we reduced the number of perturbation targets by excluding genes that are essential (associated reactions are required for growth) which can be found using FBA with GPR associations. We also performed flux variability analysis (FVA) [31] to exclude genes that are inactive (associated with reactions that cannot carry flux). These essential and inactive genes were also excluded from the analysis of dual variable ranges described above.

#### Solving successive problems iteratively

Fourth, we used an iterative algorithm by solving successive problems to optimality with increasing numbers of allowed gene deletions (k) where the solution from the previous problem (p^k^) is used as a starting point for the next problem (p^k+1^). Unlike a local search where the next solution is constrained to keep parts of the previous solution, here the next solution is not at all constrained by this starting point, but it facilitates the search by providing a good feasible solution that can be used to prune large numbers of suboptimal solutions. The successive runs improved solver stability for some difficult cases.

While all four steps were taken, we found that major runtime performance improvements were made when the bounds on dual variables and the penalty for gene deletions were applied simultaneously. We found that placing [−1, 1] bounds on the dual variables for reaction removals was very effective for the OptORF cases examined here, but these values may need to be adjusted for other models or conditions. The optimization problems were solved using CPLEX 11.2 accessed via GAMS on a linux machine with Intel Xeon 2.66GHz processors.

### SimOptStrain – simultaneous gene deletion and non-native reaction addition

SimOptStrain was developed to simultaneously consider gene deletions in a host organism and reaction additions from a universal database (see Figure 1 and Text S1 for complete mathematical formulation). Conceptually, adding a non-native reaction to a host network is equivalent to adding all non-native reactions to the host network and then deleting all non-native reactions except the desired addition. Binary variables were used in the outer problem to indicate whether non-native reactions were added (1) or not (0).

#### SimOptStrain formulation

First, GPR associations and gene deletion constraints (Equations B.16–B.20 in Text S1) were introduced to consider gene deletions instead of reaction deletions as described previously [25]. Second, the inner problem was modified to account for the addition of non-native reactions. When a non-native reaction is added, a new primal variable and a corresponding dual constraint are introduced (Equations B.2, and B10–B12). If the added reaction is irreversible, a primal constraint for non-negativity and a non-negative dual variable were also introduced (Equations B.5 and B.14). Third, new binary variables were used in the outer problem to determine whether a non-native reaction is added to a host model (Equations B.5 and B.6). A new penalty (β) for each reaction addition was applied in the outer objective function (Equation B.1), and the total number of non-native reactions added to a host model was limited to a desired value (Equation B.21). The size of such an optimization problem is generally very large due to the number of reactions in a universal database (∼4,000), but the MIP techniques described in the previous section allowed for a fast and effective solution process.

#### Metabolic model and universal reaction database

The curated KEGG [26] universal reaction database and reaction reversibility from previous studies [21], [32], and the *i*JR904 metabolic model of *E. coli* [33] were used in this work (see Dataset S1 and S2 for corresponding network details in SBML format). We excluded from consideration the reactions that cannot carry flux in a glucose aerobic environment by performing FVA with the *E. coli* model augmented with non-native reactions in the universal database. Reactions in the universal database that exist in the *E. coli* model were also not considered as additions. After this preprocessing, there were reactions which no longer exist in the current KEGG database or have the wrong directionality, and these reactions were excluded from consideration as they were found in SimOptStrain calculations (see Table S1 for the list of reaction changes to the universal database).

### BiMOMA – bi-level MIQCP approach with MOMA inner objective function

We also developed a bi-level MIQCP approach that, for the first time, uses MOMA as an inner objective problem (see Figure 2 and Text S1 for complete mathematical formulation). MOMA is a convex quadratic program (QP) that minimizes the Euclidean norm of flux changes between the wildtype and knockout strain. Here, we show how the MOMA inner problem can be replaced with its optimality conditions using complementarity [34] or strong duality [35] (Equations C.2–C.9 in Text S1) to yield a single-level MIQCP problem.

#### BiMOMA formulation

First, the MOMA inner problem is converted into a standard QP form (Equations C.2–C.4, and the left hand side of Equation C.9 in Text S1), and the dual QP of MOMA is constructed from its Lagrangian (Equations C.5–C.8, and the right hand side of Equation C.9). To enforce optimality, the complementarity conditions can be implemented in the outer problem by introducing binary variables which ensure at least one of each primal-dual constraint pair holds at equality. However, this results in a large number of additional binary variables that is not desirable. Instead, we used strong duality to set the objective values of the primal and dual pair to be equal at their optima. The quadratic equality constraint results in a non-convex region, but it can be replaced with a convex inequality constraint (Equation C.9) because the opposite inequality holds from weak duality. The resulting bi-level problem is converted into a single-level MIQCP problem, and can be directly solved using available solvers such as CPLEX.

#### Tightening the bounds on dual variables for the MOMA inner problem

While a global optimum can be obtained since the inner MOMA problem is convex, the BiMOMA problem for a genome-scale model can be very difficult to solve due to its size and non-linearity. Therefore, we investigated the dual QP of MOMA using a similar sampling procedure described in the first subsection of Materials and Methods. We modified the methods for the quadratic inner objective by simply solving dual QPs of MOMA to obtain the values of dual variables for reaction removal constraints, since the optimal solution of a convex QP is unique. Figure S2A shows the average and standard deviation of dual variable values (y-axis in log-scale) for each reaction (x-axis), and Figure S2B shows the minimum and maximum of dual variable values (y-axis in log-scale) for each reaction (x-axis) across the 1,000,000 samples of 10 gene knockouts in glucose aerobic conditions using the *i*JR904 metabolic model of *E. coli* (see Figure S2 for other conditions). Most of the shadow prices were between [−100, 100] except for a few reactions involved in cell envelope biosynthesis. We subsequently tightened the bounds on the dual variables for reaction removal constraints to be [−100, 100] (Equation C.7 and C.15) and applied a very small penalty (γ = 1e-6) to the squared Euclidean norm of these dual variables in the outer objective function (Equation C.1). The additional penalty term was found to be very effective in improving the performance of the bi-level optimization when combined with the bounds on the dual variables. The solutions from the bi-level problems were verified by solving subsequently MOMA with the identified gene deletions.

## Results

### Performance of the developed MIP techniques using OptORF

**Figure 4:**
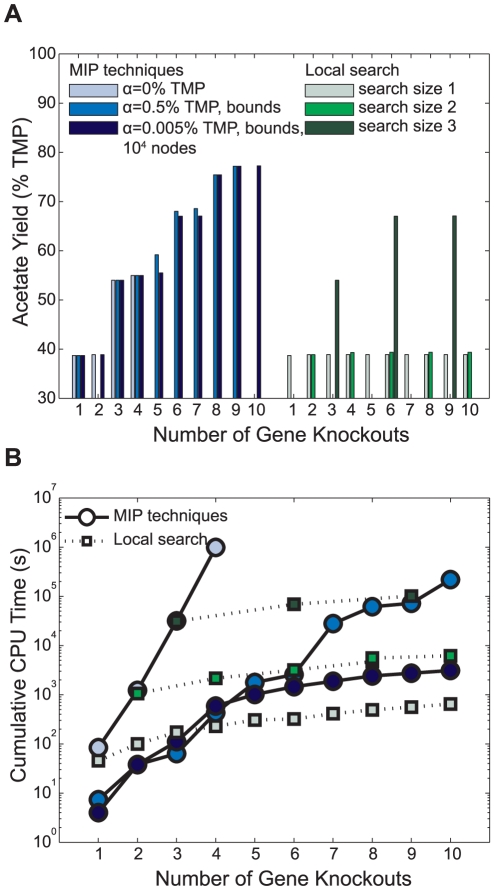
Performance of different search methods. (**A**) Predicted acetate production yields in glucose anaerobic conditions for *E. coli* strains designed using our MIP techniques or a local search method. (**B**) Cumulative CPU times for our MIP techniques (○) and a local search method (□). For both panels, cases using α = 0% TMP (light blue) or 0.5% TMP (blue) were solved to optimality and α = 0.005% TMP (dark blue) was solved with a node limit of 10^4^ (TMP = 2.56 mol acetate produced/mol glucose consumed).

We first tested the performance of the developed MIP techniques to identify gene deletion strains that are predicted to have high acetate production (Table 1) under glucose anaerobic conditions with a minimum growth rate of 0.01 h^−1^ using the *i*AF1260 metabolic model of *E. coli* [29]. We compared solutions and CPU times with and without these techniques, and to other available methods (Figure 4A and 4B), for strategies with different numbers of gene knockouts (k). First, we identified globally optimal solutions for k = 1 to 4 without using the bounds on dual variables and penalty (α = 0% TMP). The problems for k>4 could not be solved to optimality within ∼10 days. Then, we solved the problems to optimality from k = 1 to 10 using dual variable bounds and a penalty of 0.5% of the theoretical maximum production (TMP) (α = 0.5% TMP, bounds). Solutions found using the penalty and bounds were identical to the globally optimal solutions (those found without penalties and bounds) for k = 1, 3, and 4, but the CPU times were significantly lower (e.g., 10^6^ seconds versus 10^3^ seconds for k = 4). No solutions were found for 2 and 10 gene deletions because deleting an additional gene (over a 1 and 9 deletion strategy) did not increase TMP more than 0.5%, the penalty for the additional deletion. We subsequently limited the size of search tree (using the nodelim CPLEX option) to 10^4^ nodes in order to evaluate if we could identify solutions of high quality faster using multiple runs, in this case lowering the penalty (α = 0.005% TMP, bounds, 10^4^ nodes). This resulted in the same optimal solutions as those found in α = 0.5% TMP case for k = 1, 3, 4, 8, and 9, near optimal solutions that were still within 4% TMP of the optimal solutions for k = 5 to 7, and new solutions for k = 2 and 10 due to the smaller penalty. Overall, the process took less than 1 hour to find all 10 strategies. We did not observe any cases where bounding the dual variables prevented us from finding the global solutions (for k = 1 to 4) or affected the predicted growth and production rates for all k, which was confirmed by solving just the FBA inner problems after the deletions were identified. We also performed a sensitivity analysis on these bounds on dual variables by collecting optimal OptORF solutions for k = 1 to 4 with increasing restrictions on the bounds on dual variables (Figure S3), and found that the optimal solutions were only affected when the bounds were narrower than [−0.01, 0.01].

**Table 1:** Best gene deletion strategies identified by OptORF using our MIP techniques for acetate production under glucose anaerobic condition.

k	Identified Genes										Changes^a^	Yield^b^ (%)
1	*eno*											38.67
2	*eno*	*glyA*									1	38.86
3	*adhE*	*mhpF*	*ydfG*								5	53.97
4	*adhE*	*mhpF*	*ydfG*	*pgi*							1	54.93
5	*adhE*	*frmA*	*adhP*	*pgi*	*atpC*						5	59.16
6	*fsaA*	*fsaB*	*zwf*	*ldhA*	*dld*	*tpiA*					11	68.00
7	*fsaA*	*fsaB*	*zwf*	*ldhA*	*dld*	*tpiA*	*serB*				1	68.56
8	*adhE*	*mhpF*	*frmA*	*ldhA*	*dld*	*adhP*	*nuoN*	*gldA*			11	75.42
9	*adhE*	*mhpF*	*frmA*	*ldhA*	*dld*	*adhP*	*nuoN*	*mgsA*	*pgi*		3	77.15
10	*adhE*	*mhpF*	*frmA*	*ldhA*	*dld*	*adhP*	*nuoN*	*mgsA*	*gdhA*	*ptsH*	3	77.25

a ‘Changes’ refer to the number of genes which are newly introduced in the solution with k deletions or removed from the solution with k–1 deletions.

b Yield is reported as % of the TMP for wildtype strain with a maximum glucose uptake rate of 10 mmol gDW^−1^ h^−1^ (2.56 mol acetate produced/mol glucose consumed). gDW stands for gram dry weight.

To compare the proposed MIP techniques to local search methods, we implemented and modified the Genetic Design through Local Search algorithm (GDLS [28]) to use gene deletions instead of unique manipulations, where the latter can be comprised of multiple gene deletions. GDLS was performed with local search sizes from 1 to 3, where a local search size of *n* indicates that a total of *n* genes were removed from or added to the previous strategy. While the computational requirements of our MIP techniques and local search methods were comparable, in many cases the local search (GDLS) was unable to find better deletion strategies (Figure 4). This is because the best strategies found using our method did not share a significant number of genes with simpler strategies (Table 1). For example, none of the gene deletions in the k = 5 strategy were found in the k = 6 strategy indicating a local search size of 11 would be needed to find it. We also tested the performances of cellular genetic, evolutionary, and simulated annealing algorithms in OptFlux v2.1 [36] using a maximum of 10 gene deletions and 50,000 function evaluations (∼6.5×10^4^ seconds). These algorithms found strains with lower acetate production (∼40% TMP, data not shown).

**Figure 5:**
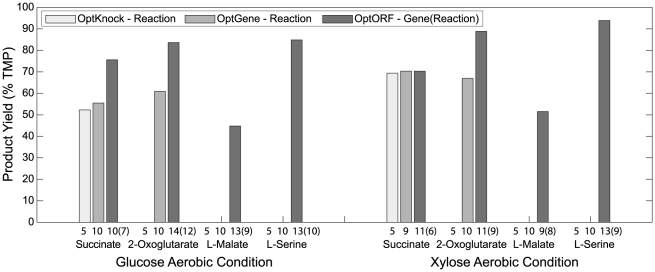
Product yields for *E. coli* strains designed to generate different products. Different colors indicate OptKnock (light grey), OptGene (grey), and OptORF (dark grey). The numbers on the x-axis correspond to the number of reaction deletions identified (or maximum allowed if no strategy was found) by OptKnock and OptGene [30], or gene deletions (equivalent reaction deletions listed in parentheses) identified by OptORF (this study). The product yields for OptKnock and OptGene were taken from an earlier study [30] and re-calculated based on TMP values without a minimum growth requirement. A missing bar indicates that no strategy was previously found.

We additionally used OptORF to find high production strategies for metabolites that were previously found to be difficult to couple to biomass production under glucose and/or xylose aerobic conditions (Figure 5) [30]. The OptKnock [19] and OptGene [17] results shown in Figure 5 are from a recent study where strategies had a maximum of 5 or 10 reaction deletions, respectively [30]. For the OptORF cases, the model and simulation conditions from the earlier study [30] were used to obtain the results shown in Figure 5 (including minimum growth rate requirement, maximum substrate uptake rates, metabolic network changes, and ‘tilting’ of the inner objective function – which helps eliminate strategies where alternate maximal growth solutions with high and low productivity are possible). For comparison purposes, we identified the number of reaction deletions that are equivalent to each OptORF gene deletion strategy. Overall, our methods found strategies with higher production using similar numbers of deletions, and also identified strategies for cases where other approaches could not (missing bars in Figure 5), including malate and serine.

### Identification of novel enzyme additions using SimOptStrain

To demonstrate the benefit of considering gene deletions and non-native reaction additions simultaneously, we applied the SimOptStrain approach to succinate and glycerol production under glucose aerobic conditions with a minimum growth of 0.1 h^−1^. We used a metabolic model of *E. coli* with GPR associations [33] and a curated KEGG universal database that was used in the previous OptStrain study [21]. Even after preprocessing, the size of the problem involving addition of multiple non-native reactions was still very large. However, when applied to SimOptStrain, the MIP solution techniques resulted in significant reductions in computational requirements (e.g., from ∼15 CPU hours to 0.4 CPU hours for succinate production considering strategies with 3 gene deletions (k = 3) and 1 non-native reaction addition (k′ = 1)). We focused here on finding strategies with improved product yields rather than evaluating their relative optimality (as was done above for OptORF). The best gene deletion strategies without any addition of non-native reactions were identified using OptORF, and the resulting solutions were compared to new strategies which give higher yields with the same number of deletions but with non-native reaction additions. First, a high penalty value for each addition (β = 10% TMP of wildtype) and deletion (α = 1% TMP) was applied to find strategies with significantly improved yields. Second, a lower value of the reaction addition penalty (β = 1% TMP of wildtype and α = 1%) was used to identify additional strategies which may further improve the yields. In addition, ‘tilting’ of the inner objective function [30] was employed to eliminate strategies where alternate solutions with high and low productivity are possible.

**Figure 6:**
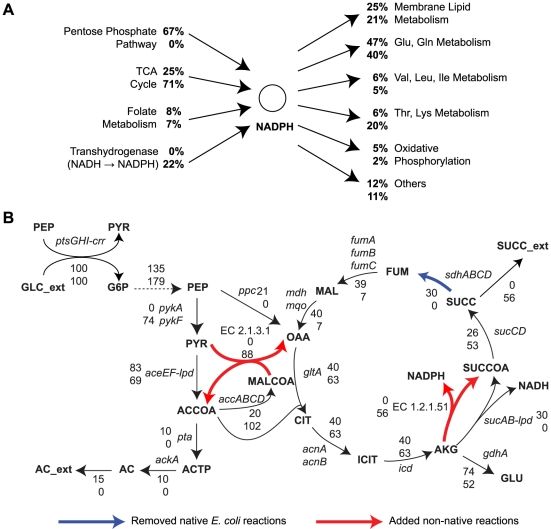
Fluxes involving NADPH production/consumption and central metabolism. The top numbers are for wild-type and the bottom numbers are for a predicted succinate producing strain (*ΔsdhC Δgnd ΔglyA*+EC 1.2.1.52+EC 2.1.3.1 reactions). (A) Metabolic pathways producing or consuming NADPH are shown. The numbers are percentages of the total NADPH produced or consumed, where 100% is 15.7 mmol gDW^−1^ h^−1^ for wild-type (first line) and 7.3 mmol gDW^−1^ h^−1^ for succinate producing strain (second line). gDW stands for gram dry weight. Abbreviations of metabolites: Glu, glutamate; Gln, glutamine; Ile, isoleucine; Leu, leucine; Lys, lysine; Thr, threonine; Val, valine. (B) Metabolic fluxes and genes associated with each reaction in the central metabolic networks are shown. Blue arrows indicate removed native *E. coli* reactions, and red arrows indicate added non-native reactions. The numbers are relative fluxes normalized with respect to the total glucose uptake rate (100% is 10 mmol glucose gDW^−1^ h^−1^). Abbreviations of metabolites (‘_ext’ indicates extracellular): AC, acetate; ACCOA, acetyl-CoA; ACTP, acetyl phosphate; AKG, 2-oxoglutarate; CIT, citrate; FUM, fumarate; G6P, glucose 6-phosphate; GLC, glucose; ICIT, isocitrate; MAL, malate; MALCOA, malonyl-CoA; OAA, oxaloacetate; PEP, phosphoenolpyruvate; PYR, pyruvate; SUCC, succinate; SUCCOA, succinyl-CoA.

For succinate production, we found that there are no non-native reactions which improve the TMP when added to the wildtype *E. coli* model. Therefore, the previous multi-step OptStrain procedure would not identify any non-native reactions from the KEGG database to be added to the host model. However, when we explored the simultaneous deletion of genes in *E. coli* model and addition of non-native reactions from KEGG database using SimOptStrain, we were able to identify non-native reactions which can significantly improve the amount of succinate produced when the *E. coli* mutant strains achieve their maximum growth rate (Table 2). Without the addition of identified reactions, these mutant strains would not produce succinate or would exhibit a significantly lower level of succinate production at the maximum growth (data not shown). A common characteristic of the identified non-native reaction additions was the use of NADP(H) instead of NAD(H) cofactors (Figure 6A). The reactions associated with enzyme commission (EC) numbers 1.2.1.51 and 1.2.1.52 produce NADPH as a cofactor and replace native *E. coli* reactions which instead produce NADH. Additionally, reactions catalyzed by EC 1.4.1.9 and 1.4.1.20 enzymes convert carboxylates and ammonia into amino acids using NADH, and the amine groups from these amino acids were transferred onto 2-oxoglutarate to produce glutamate using different transaminases. This reduces the flux through glutamate dehydrogenase, which uses NADPH to convert ammonia and 2-oxoglutarate into glutamate. This additional NADPH (from reduced glutamate dehydrogenase flux) lowered fluxes in the pentose phosphate pathway and increased fluxes in the TCA cycle thereby improving succinate production (Figure 6B). Addition of another non-native reaction associated with EC 2.1.3.1 further increased fluxes in the TCA cycle by reducing the amount of acetate secreted as a by-product.

**Table 2:** Gene deletion and reaction addition strategies identified by SimOptStrain for succinate production under glucose aerobic condition.

k	Deleted Genes					k′	Added Reactions (EC No.^a^)		Growth Rate (h^−1^)	TMP^b^ (mol/mol)	Yield^c^ (%)
	wildtype								0.88	1.5	0.0
3	*sdhC*	*pta*	*eutD*			0	None^d^		0.83	1.5	8.8
	*sdhC*	*gnd*	*glyA*			1	1.2.1.52		0.62	1.5	32.5
	*sdhC*	*gnd*	*glyA*			2	1.2.1.52	2.1.3.1	0.62	1.5	37.3
4	*sdhC*	*gnd*	*glyA*	*pntA*		0	None^d^		0.59	1.5	38.2
	*cyoA*	*cydA*	*adhE*	*pntA*		1	1.2.1.51		0.17	1.5	60.4
5	*cyoA*	*cydA*	*lpd*	*ptsH*	*atpA*	0	None^d^		0.11	1.5	54.4
	*cyoA*	*cydA*	*adhE*	*ptsH*	*atpA*	1	1.4.1.20		0.12	1.5	67.5
	*cyoA*	*cydA*	*adhE*	*ptsH*	*atpA*	1	1.4.1.9		0.12	1.5	67.5

a Enzyme Commission number.

1.2.1.52 2-Oxoglutarate+CoA+NADP^+^< = >Succinyl-CoA+CO_2_+NADPH.

2.1.3.1 Malonyl-CoA+Pyruvate< = >Acetyl-CoA+Oxaloacetate.

1.2.1.51 Pyruvate+CoA+NADP^+^< = >Acetyl-CoA+CO_2_+NADPH.

1.4.1.20 L-Phenylalanine+H_2_O+NAD^+^< = >Phenylpyruvate+NH_3_+NADH.

1.4.1.9 L-Valine+H_2_O+NAD^+^< = >3-Methyl-2-oxobutanoate+NH_3_+NADH.

b Theoretical maximum production (TMP) is reported as mol succinate produced/mol glucose consumed for each strain with non-native reaction additions, but without gene deletions. A maximum glucose uptake rate of 10 mmol gDW^−1^ h^−1^ and a maximum oxygen uptake rate of 18.5 mmol gDW^−1^ h^−1^ were used.

c Yield is reported as % of the TMP for each strain after reactions are added.

d Best strategies without addition of non-native reactions.

For glycerol production, the addition of non-native reactions from the KEGG database could improve the TMP up to ∼220% of the TMP for the wildtype *E. coli* strain. Interestingly, the non-native reactions we found using SimOptStrain yielded a suboptimal increase in the TMP (140%∼170% of the wildtype TMP, Table 3). There were numerous combinations of non-native reactions which yielded the maximum increase in TMP (∼220%); thus it would be almost impossible to identify these suboptimal non-native reactions using the previous OptStrain procedure. One of the most frequently identified reactions was associated with EC 3.1.3.21, which dephosphorylates glycerol-3-phosphate into glycerol. The addition of these non-native reactions and deletion of ∼5 to 6 *E. coli* genes were predicted to significantly improve glycerol production when coupled to the growth (up to ∼106% TMP of wildtype). Without the addition of the identified non-native reactions, we found that the best strategies using only gene deletions resulted in very low glycerol yields (less than 0.1% TMP for 3 gene knockout mutants and 6.8% TMP for 6 gene knockout mutants, see Table 3).

**Table 3:** Gene deletion and reaction addition strategies identified by SimOptStrain for glycerol production under glucose aerobic condition.

k	Deleted Genes						k′	Added Reactions (EC No.^a^)		Growth Rate (h^−1^)	TMP^b^ (mol/mol)	Yield^c^ (%)
	Wildtype									0.88	0.91	0.0
3	*glpK*	*frmA*	*gldA*				0	None^d^		0.88	0.91	0.06
5	*pgk*	*fbp*	*gloB*	*nuoN*	*gldA*		1	3.1.3.21		0.21	1.51	47.1
	*pgk*	*fbp*	*gloB*	*nuoN*	*pgi*		1	3.1.3.21		0.21	1.51	55.1
	*pgk*	*fbp*	*gloA*	*frmA*	*gldA*		1	2.7.1.142		0.35	1.58	59.3
	*pgk*	*fbp*	*gloA*	*frmA*	*gldA*		1	3.1.3.21		0.30	1.51	62.6
6	*fsaA*	*fsaB*	*gloB*	*tpiA*	*eda*	*deoC*	0	None^d^		0.64	0.91	6.8
	*pgk*	*fbp*	*gloB*	*nuoN*	*gldA*	*cyoA*	2	3.1.3.21	2.1.3.1	0.17	1.51	64.1

a Enzyme Commission number.

3.1.3.21 sn-Glycerol 3-phosphate+H_2_O< = >Glycerol+Orthophosphate.

2.7.1.142 sn-Glycerol 3-phosphate+D-Glucose< = >Glycerol+D-Glucose 6-phosphate.

2.1.3.1 Malonyl-CoA+Pyruvate< = >Acetyl-CoA+Oxaloacetate.

b Theoretical maximum production is reported as mol glycerol produced/mol glucose consumed for each strain with non-native reaction additions, but without gene deletions. A maximum glucose uptake rate of 10 mmol gDW^−1^ h^−1^ and a maximum oxygen uptake rate of 18.5 mmol gDW^−1^ h^−1^ were used.

c Yield is reported as % of the TMP for each strain after reactions are added.

d Best strategies without addition of non-native reactions (no strategy was found for k = 5 and k′ = 0 because any small production increases (over the k = 3 strategy) were negated by the penalty α = 10^−6^).

### Un-evolved strain designs using BiMOMA

**Figure 7:**
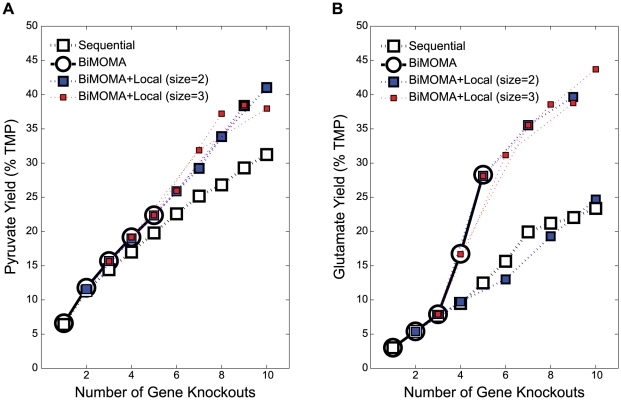
Improvements in product yields in glucose aerobic conditions for *E. coli* strains designed using BiMOMA. (A) Pyruvate and (B) Glutamate. The best BiMOMA strategies (○) were identified for k = 1 to 5 using a penalty of 0.5% TMP, and were combined with a local search (□) with search sizes of 2 or 3. BiMOMA+local search size of 2 starts from the best BiMOMA solutions for k = 2 and 3; and BiMOMA+local search size of 3 starts from the best BiMOMA solutions for k = 3, 4, and 5. A sequential search was also performed, which is a local search with search size of 1 starting from the best k = 1 solution.

To find ‘un-evolved’ *E. coli* strain designs for improving biochemical production we used BiMOMA, the first MIQCP bi-level strain design approach that uses a quadratic inner problem. For the ‘un-evolved’ strain designs, biochemical production does not have to be coupled to the cellular growth in the mutant strain. Instead production is improved when the metabolic fluxes are re-adjusted after a gene(s) is deleted. These adjusted fluxes can be predicted by finding solutions that are closest to the wildtype flux distribution. We used a metabolic model of *E. coli* with GPR associations [33] to identify optimal gene deletion strategies that would immediately improve production of pyruvate or glutamate in a glucose aerobic condition (Figure 7). The same penalty for each additional deletion (α = 0.5% TMP), minimum growth rate of 0.1 h^−1^, and bounds on the dual variables [−100, 100] were used for all cases. First, we identified the best strategies for 1 to 10 deletions (k = 1 to 10) using a local search with search size of 1, which is equivalent to a sequential search (labeled as Sequential in Figure 7). Next, we solved the problems using our BiMOMA approach to optimality from k = 1 to 5 (labeled as BiMOMA in Figure 7). The MIP solution techniques also significantly reduced the solution times for this quadratic bi-level problem (e.g. from ∼65 CPU hours to 2 CPU hours for pyruvate production considering strategies with 3 gene deletions). A sensitivity analysis on the bounds on dual variables was also performed to check whether these bounds prevented finding optimal solutions for k = 1 to 3 by changing the bounds from [−infinity, +infinity] to [−10, 10] (Figure S3). We did not observe cases where the bounds of [−100, 100] prevented us from finding the optimal solutions. To find more complex strategies for k = 6 to 10, we also combined BiMOMA with a local search method by applying a local search with search size of 2 using the BiMOMA solutions for k = 2 and 3 as starting points (labeled as BiMOMA+Local, size = 2 in Figure 7), and a local search with search size of 3 using the BiMOMA solutions for k = 3, 4 and 5 as starting points (labeled as BiMOMA+Local, size = 3). The combined BiMOMA and local search resulted in strain designs with significantly higher product yields than the sequential method (by up to ∼10% higher TMP for pyruvate and ∼20% higher TMP for glutamate).

In the pyruvate case, the differences in yields between the sequential search and BiMOMA search were somewhat moderate for k = 1 to 5, but the sequential search missed higher production strategies as the number of allowed gene deletions increased (Figure 7A). These results indicate that using a bigger search size is advantageous, which can be explained by significant changes in genes that need to be deleted (Table 4). However, the best strategy for k = 10 was found during the BiMOMA+Local search of size 2. This is due to the fact that more genes in the best strategy for k = 10 were shared by the strategies found during the search path of size 2 than by the strategies found during the path of size 3.

**Table 4:** Top gene deletion strategies identified by BiMOMA (k<5) or BiMOMA+Local Search (k>5) for pyruvate production under glucose aerobic condition.

k	Identified Genes										Changes^a^	Yield^b^ (%)
1	*aceE*											6.59
2	*lpd*	*gnd*									3	11.78
3	*lpd*	*gnd*	*brnQ*								1	15.69
4	*lpd*	*gnd*	*brnQ*	*poxB*							1	19.16
5	*lpd*	*gnd*	*gdhA*	*poxB*	*ppc*						3	22.39
6	*lpd*	*gnd*	*brnQ*	*poxB*	*pps*	*mdh*					5	25.87
7	*lpd*	*gnd*	*brnQ*	*poxB*	*gdhA*	*lysP*	*pgi*				5	31.86
8	*lpd*	*gnd*	*brnQ*	*poxB*	*gdhA*	*ppc*	*pgi*	*purT*			3	37.21
9	*lpd*	*gnd*	*brnQ*	*poxB*	*gdhA*	*pps*	*mdh*	*pfkA*	*pfkB*		7	38.46
10	*lpd*	*gnd*	*brnQ*	*poxB*	*gdhA*	*pps*	*mdh*	*pfkA*	*pfkB*	*mqo*	1	41.03

a ‘Changes’ refer to the number of genes which are newly introduced in the solution with k deletions or removed from the solution with k–1 deletions.

b Yield is reported as % of the TMP (2 mol pyruvate produced/mol glucose consumed) for wildtype strain with a maximum glucose uptake rate of 10 mmol gDW^−1^ h^−1^ and a maximum oxygen uptake rate of 18.5 mmol gDW^−1^ h^−1^.

The benefit of a bigger search size is more evident in the glutamate case (Figure 7B). The changes in identified genes were not as remarkable as the pyruvate case (Table 5), but the consequence of these small gene differences was more striking. For example, a significant improvement in yields from k = 3 to 4 and 4 to 5 was found using BiMOMA (8.8% and 11.6% TMP), while only a small increase was shown by the sequential search (1.7% and 2.4% TMP). Surprisingly, the combined BiMOMA and local search with search size of 2 resulted in lower yields than those found using the sequential search for k = 6 and 8, but identified a strategy with higher predicted yield for k = 10.

**Table 5:** Top gene deletion strategies identified by BiMOMA (k<5) or BiMOMA+Local Search (k>5) for glutamate production under glucose aerobic condition.

k	Identified Genes										Changes^a^	Yield^b^ (%)
1	*sucA*											3.01
2	*sucA*	*kgtP*									1	5.41
3	*sdhC*	*kgtP*	*dcuC*								3	7.90
4	*sdhC*	*kgtP*	*dcuC*	*gadC*							1	16.74
5	*sdhC*	*kgtP*	*dcuC*	*gadC*	*gnd*						1	28.29
6	*sdhC*	*kgtP*	*dcuC*	*gadC*	*gnd*	*pntB*					1	31.16
7	*sdhC*	*kgtP*	*dcuC*	*gadC*	*gnd*	*brnQ*	*ptsH*				3	35.54
8	*sdhC*	*kgtP*	*dcuC*	*gadC*	*gnd*	*brnQ*	*ptsH*	*citF*			1	38.56
9	*sdhC*	*kgtP*	*dcuC*	*gadC*	*gnd*	*brnQ*	*ptsH*	*tpiA*	*fabH*		3	39.65
10	*sdhC*	*kgtP*	*dcuC*	*gadC*	*gnd*	*brnQ*	*ptsH*	*citF*	*pta*	*eutD*	5	43.70

a ‘Changes’ refer to the number of genes which are newly introduced in the solution with k deletions or removed from the solution with k–1 deletions.

b Yield is reported as % of the TMP (1.15 mol glutamate produced/mol glucose consumed) for wildtype strain with a maximum glucose uptake rate of 10 mmol gDW^−1^ h^−1^ and a maximum oxygen uptake rate of 18.5 mmol gDW^−1^ h^−1^.

Using the BiMOMA approach, we were able to efficiently identify production strategies with up to 40–45% theoretical maximum yields for glutamate and pyruvate (Figure 7), while the existing genetic algorithm based approach OptGene only found strategies with 2–5% of the maximum yields using a maximum of 10 knockouts and 100,000 function evaluations (data not shown). These results illustrate the advantages of using mixed-integer programming to solve bi-level problems as they can significantly reduce solution times while still finding high production strategies.

## Discussion

The use of computational approaches in metabolic engineering has grown rapidly, alongside an increasing number of genome-scale metabolic models [2], [37], [38], [39], [40]. These models can provide detailed predictions regarding metabolic flux distributions and identify strains with enhanced biochemical production. The computational models can predict the effects of genetic modifications (including gene knockout, gene overexpression, or gene/reaction addition), and they can be used to identify the best set(s) of strain modifications to improve production by considering all possible modifications. Computational approaches have the capability to generate a diverse collection of modification strategies that can be tested experimentally. In this study, we presented two new strain design approaches and mixed-integer programming techniques which allow us to solve different types of strain design problems more effectively, thereby facilitating the strain design process.

The MIP techniques developed in this study can be applied to most existing bi-level approaches for strain design, synthetic lethal identification, or network identification [2], [41], [42]. We demonstrated this by applying the developed techniques to OptORF and SimOptStrain, both of which use the optimal cellular growth as an underlying assumption for predicting mutant phenotypes. The results from the OptORF case show how these techniques can significantly improve the performance of the strain design approaches, thereby allowing us to more quickly identify perturbation strategies with large numbers of modifications. This alleviates one of the major limitations in the current strain design process, and provides us with more options that can be explored experimentally. An important step when using these techniques is finding appropriate bounds for the dual variables, since bounds that are too restrictive may prevent the optimal solutions from being found. The sampling procedure used here is one way to approximate the dual bounds, and this should be done before applying the developed approaches to other models or growth conditions.

With these runtime performance improvements, the SimOptStrain approach can now be used to simultaneously consider the deletion of genes in a host organism and addition of non-native reactions. The simultaneous search broadens the scope of strain designs and identifies novel combinations of modifications, which could not have been found previously using a multi-step procedure. In addition to strain design, SimOptStrain could also be used to refine models (by adding and removing reactions) for cases when FBA does not correctly predict by-product secretion. Improvements in the universal reaction database are still needed, particularly with respect to reaction reversibility which affects constraint-based model predictions [43]. In this work, we used reaction reversibility based on KEGG (see [32] for details), and found that removing strategies involving incorrect reaction directionality was more time consuming than obtaining strategies with the strain design approach itself. This issue could possibly be resolved by using a large collection of genome-scale metabolic models, which may have better curation of reaction directionality than universal databases. In addition, these models usually come with gene to protein to reaction (GPR) associations which can be used to eliminate reactions without associated genes or make the addition of reactions from a related organism preferable. In order to achieve this, common nomenclature for metabolites and reactions across models would be needed as aligning models from multiple sources is a current challenge [44], [45].

We further expanded the application of the solution techniques to BiMOMA, the first mixed-integer programming approach that uses MOMA [13] as an underlying assumption for the inner problem. Previously, OptGene [17] solved this bi-level problem using genetic algorithms, but its application to a large scale search is currently limited by the convergence of the algorithms used. Our solution techniques allowed for fast identification of metabolic engineering strategies involving a large number of gene knockouts for improving the production of different biochemicals. A number of previous studies successfully engineered microbial strains using the MOMA assumption [15], [16], [18], but they employed a sequential search or considered only a small number of modifications. A sequential search may identify an optimal strategy involving a few modifications, but it is more likely to converge to sub-optimal strategies as the number of modifications increases (Figure 7). One may argue that a large number of genetic modifications would not be necessary and a few key modifications would be sufficient. However, a lot of metabolic engineering successes required a large number of perturbations involving gene deletion, gene overexpression, or gene addition [15],[46],[47]. As new computational strain design approaches are rapidly being developed to account for these different types of perturbations [13], [14], [21], [22], [24], [25], [36], [48], [49], the solution techniques used in this study would benefit approaches that use a bi-level architecture to enumerate mutants with desired phenotypes.

In summary, we developed two new bi-level strain design approaches using mixed-integer programming. The developed approaches could be useful particularly for identifying novel metabolic engineering strategies to improve production of non-native secondary metabolites. We also presented mixed-integer programming solution techniques based on concepts from duality to effectively identify genetic perturbation strategies, within a reasonable amount of time even for a large number of perturbations. The MIP techniques were successfully applied to existing strain design approaches as well as new approaches developed in this study. They will likely improve the efficiency of other bi-level problems as well, including model identification, synthetic lethal identification, and objective function prediction [41], [42], [50], [51]. We believe these approaches and techniques will contribute to the field of metabolic engineering by accelerating the strain design process.

## Supporting Information

Figure S1 **Analysis of dual variables for reaction removals using dual LP of FBA in different media conditions.** Results from sampling of dual variable values are shown for (A) glucose aerobic, (B) glucose anaerobic, (C) xylose aerobic, and (D) xylose anaerobic conditions. The top plots show for each reaction the average of positive dual variable values (downward triangle) and negative dual variable values (upward triangle) observed over different samples, and their respective standard deviations (error bars). The averages and standard deviations were calculated for positive and negative values separately, and zero values were excluded from these statistical calculations. The bottom plots show the maximum (downward triangle) and minimum (upward triangle) of observed dual variable values for each reaction across the 1,000,000 samples of 10 gene knockouts in each condition.
(PDF)

Figure S2 **Analysis of dual variables for reaction removals using dual QP of MOMA in different media conditions.** Results from sampling of dual variable values are shown for (A) glucose aerobic, (B) glucose anaerobic, (C) xylose aerobic, and (D) xylose anaerobic conditions. The top plots show for each reaction the average of positive dual variable values (downward triangle) and negative dual variable values (upward triangle) observed over different samples, and their respective standard deviations (error bars). The averages and standard deviations were calculated for positive and negative values separately, and zero values were excluded from these statistical calculations. The bottom plots show the maximum (downward triangle) and minimum (upward triangle) of observed dual variable values for each reaction across the 1,000,000 samples of 10 gene knockouts in each condition.
(PDF)

Figure S3 **Sensitivity analysis of the bounds on dual variables.** Optimal solutions were collected with no bounds or different values of bounds on dual variables for (A) acetate production using OptORF and (B) pyruvate production using BiMOMA, respectively.
(PDF)

Table S1 **List of reaction changes to the universal database.** This table includes the list of reactions removed in this study from the original universal database (reported in [21]).
(XLSX)

Text S1 **Detailed formulations of the bi-level strain design approaches used in this study.** Complete formulations of single-level transformed bi-level strain design approaches are included for (A) OptORF without regulatory considerations, (B) SimOptStrain, and (C) BiMOMA.
(PDF)

Dataset S1 **iJR904 model in SBML format.** The file contains details for the iJR904 model in SBML format. Most of the compound abbreviations used in this file differ from those in the original iJR904 publication [33] and instead match those abbreviations used in the universal database (Dataset S2). There are more compounds and reactions in this SBML file than originally published [33], since some compounds in iJR904 were matched to more than one compound in the universal database.
(XML)

Dataset S2 **Universal database in SBML format.** The file contains details for the universal database used in the SimOptStrain simulations. This database was modified slightly from the original published database (reported in [21]) by specifying reaction directionality and excluding those reactions listed in Table S1 (see Materials and Methods for details).
(XML)
