# Supplementary figures and images for: Large-Scale Bi-Level Strain Design Approaches and Mixed-Integer Programming Solution Techniques
Source: PLoS One. 2011 Sep 9;6(9):e24162. doi: 10.1371/journal.pone.0024162 (PMC3175644; doi:10.1371/journal.pone.0024162)

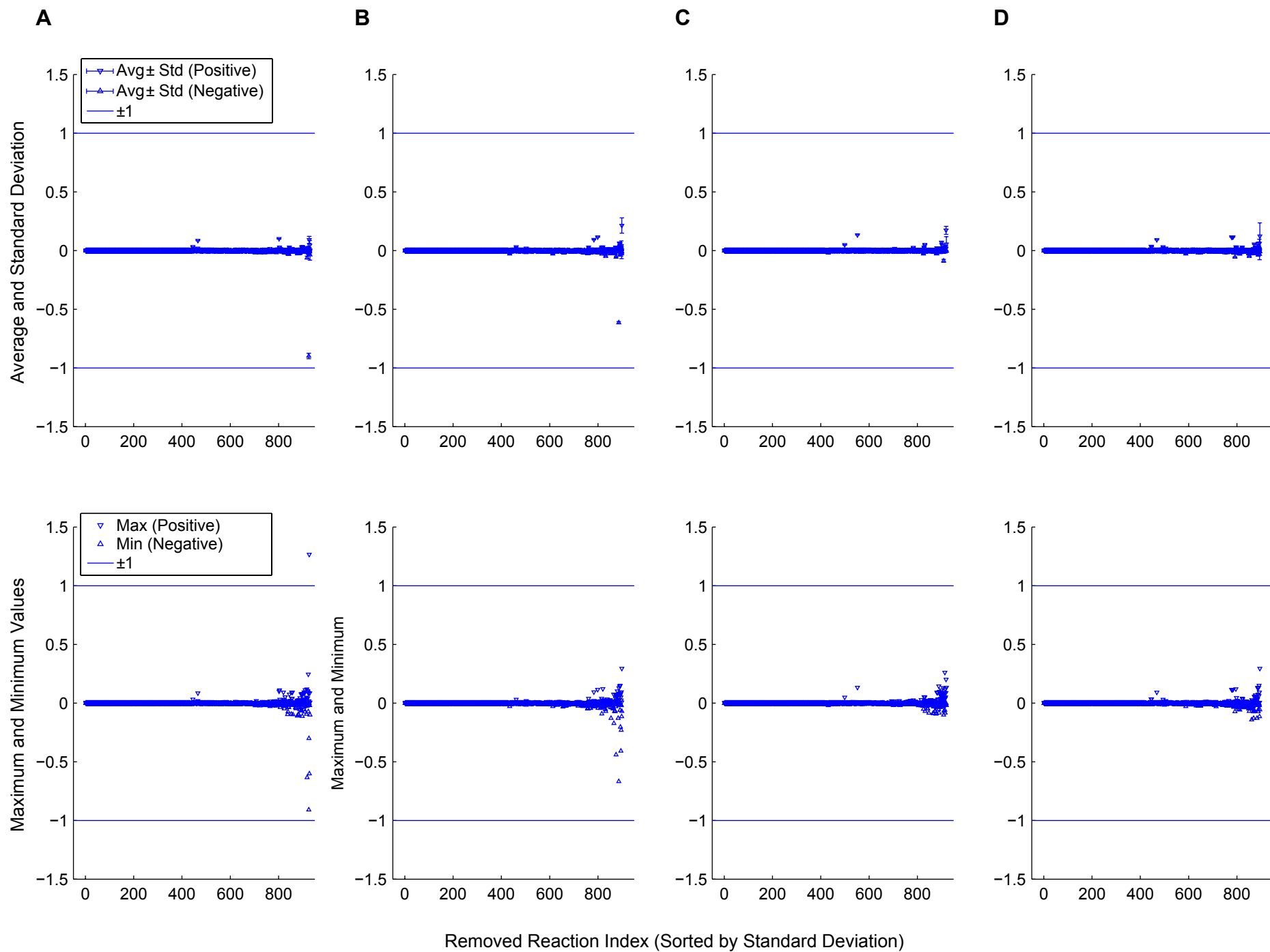

Supplement: Figure S1 — Analysis of dual variables for reaction removals using dual LP of FBA in different media conditions. Results from sampling of dual variable values are shown for (A) glucose aerobic, (B) glucose anaerobic, (C) xylose aerobic, and (D) xylose anaerobic conditions. The top plots show for each reaction the average of positive dual variable values (downward triangle) and negative dual variable values (upward triangle) observed over different samples, and their respective standard deviations (error bars). The averages and standard deviations were calculated for positive and negative values separately, and zero values were excluded from these statistical calculations. The bottom plots show the maximum (downward triangle) and minimum (upward triangle) of observed dual variable values for each reaction across the 1,000,000 samples of 10 gene knockouts in each condition. (PDF) [file pone.0024162.s001.pdf]

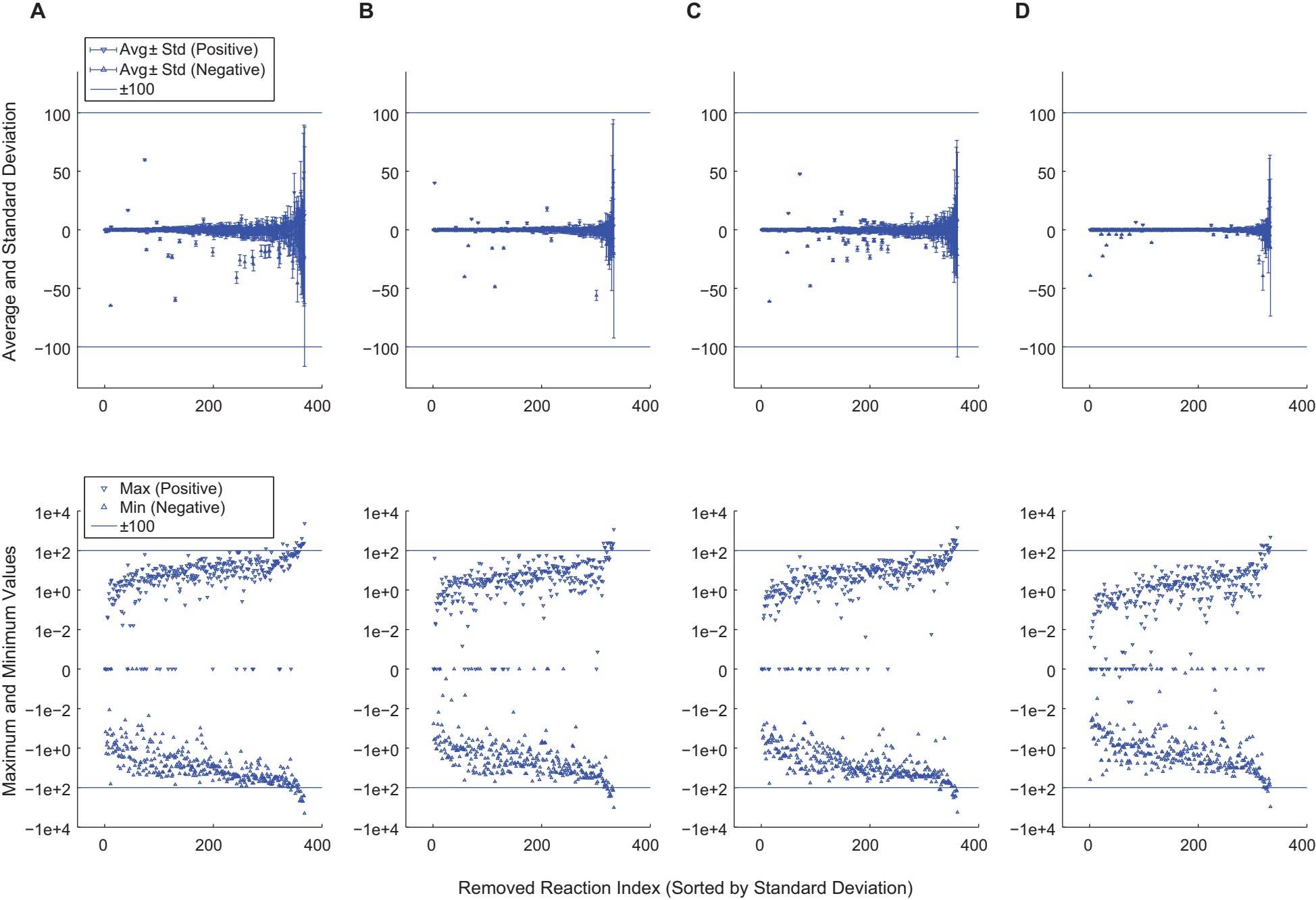

Supplement: Figure S2 — Analysis of dual variables for reaction removals using dual QP of MOMA in different media conditions. Results from sampling of dual variable values are shown for (A) glucose aerobic, (B) glucose anaerobic, (C) xylose aerobic, and (D) xylose anaerobic conditions. The top plots show for each reaction the average of positive dual variable values (downward triangle) and negative dual variable values (upward triangle) observed over different samples, and their respective standard deviations (error bars). The averages and standard deviations were calculated for positive and negative values separately, and zero values were excluded from these statistical calculations. The bottom plots show the maximum (downward triangle) and minimum (upward triangle) of observed dual variable values for each reaction across the 1,000,000 samples of 10 gene knockouts in each condition. (PDF) [file pone.0024162.s002.pdf]

A

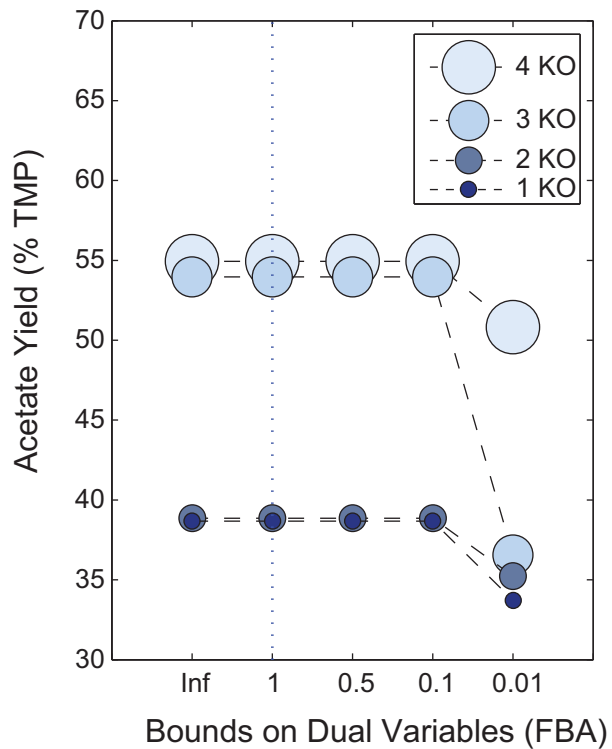

B

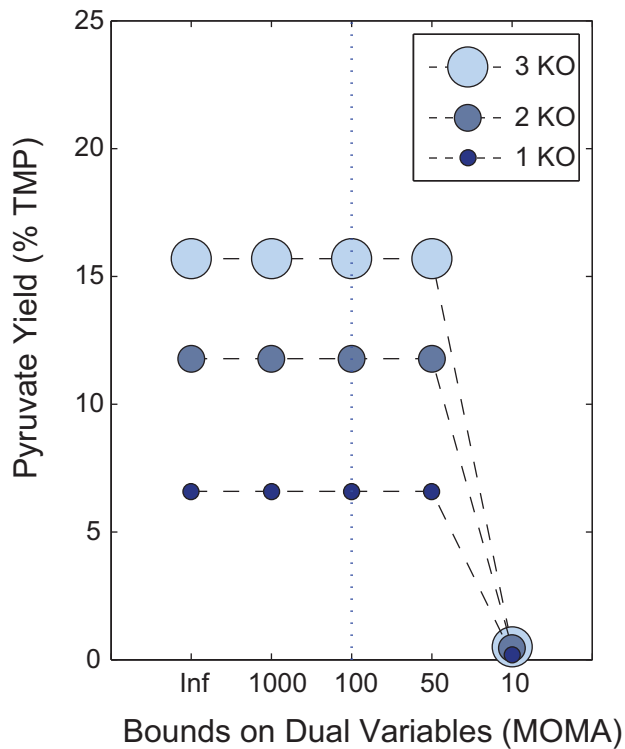

Supplement: Figure S3 — Sensitivity analysis of the bounds on dual variables. Optimal solutions were collected with no bounds or different values of bounds on dual variables for (A) acetate production using OptORF and (B) pyruvate production using BiMOMA, respectively. (PDF) [file pone.0024162.s003.pdf]
