# Supplementary material for: Large-Scale Bi-Level Strain Design Approaches and Mixed-Integer Programming Solution Techniques
Source: PLoS One. 2011 Sep 9;6(9):e24162. doi: 10.1371/journal.pone.0024162 (PMC3175644; doi:10.1371/journal.pone.0024162)
Supplement: Text S1 — Detailed formulations of the bi-level strain design approaches used in this study. Complete formulations of single-level transformed bi-level strain design approaches are included for (A) OptORF without regulatory considerations, (B) SimOptStrain, and (C) BiMOMA. (PDF) [file pone.0024162.s005.pdf]

Supporting Information Text S1

Nomenclature

i – metabolites; j – reactions; l – non - native reactions; n – enzymes; g – genes

$J_{LB} = \{ j \in J \mid \text{the flux through reaction } j \text{ has a lower bound} \}$

$L_{LB} = \{ l \in L \mid \text{the flux through non - native reaction } l \text{ has a lower bound} \}$

$J_{GPR} = \{ j \in J \mid \text{reaction } j \text{ has known GPR associations} \}$

$N(j) = \{ n \in N \mid \text{enzyme } n \text{ is associated with reaction } j \}$

$G(n) = \{ g \in G \mid \text{gene } g \text{ is associated with enzyme } n \}$

c_j – outer objective coefficient; p_j – inner objective coefficient; S_{ij} – stoichiometric coefficient

v_j – flux through reaction j; w_l – flux through reaction l; $u_i, \lambda_j, h_j, \eta_l, g_l$ – dual variables

v_j^{lb} – lower bound on v_j; w_l^{lb} – lower bound on w_l; h_j^{lb} / h_j^{ub} – lower / upper bounds on h_j

y_g / b_n / d_j – binary variables for gene / enzyme / reaction status

α – penalty for gene deletion; β – penalty for reaction addition

γ – penalty for dual variables h_j; v_j^{wt} – wildtype flux through reaction j

k – number of allowed gene deletions; k' – number of allowed reaction additions

A. OptORF without transcriptional regulatory constraints

$$\max \quad \sum_j c_j v_j - \alpha \sum_g (1 - y_g) \tag{A.1}$$

$$\text{s.t.} \quad \sum_j S_{ij} v_j = 0 \qquad \forall i \in I : (u_i) \tag{A.2}$$

$$v_j \ge v_j^{lb} \qquad if \;\; d_j = 1 \qquad \forall j \in J_{LB} : (\lambda_j) \tag{A.3}$$

$$v_j = 0 \qquad if \;\; d_j = 0 \qquad \forall j \in J : (h_j) \tag{A.4}$$

$$\sum_i S_{ji} u_i = p_j \qquad if\ d_j = 1 \qquad \forall j \in J \setminus J_{LB} \tag{A.5}$$

$$\sum_i S_{ji} u_i - \lambda_j = p_j \qquad if\ d_j = 1 \qquad \forall j \in J_{LB} \tag{A.6}$$

$$\sum_i S_{ji} u_i + h_j = p_j \qquad if\ d_j = 0 \qquad \forall j \in J \tag{A.7}$$

$$\lambda_j \geq 0 \qquad \forall j \in J_{LB} \tag{A.8}$$

$$\sum_j p_j v_j = \sum_{j \in J_{LB}} (-v_j^{lb}) \lambda_j \tag{A.9}$$

$$d_j \geq b_n \qquad \forall j \in J_{GPR}, n \in N(j) \tag{A.10}$$

$$d_j \leq \sum_{n \in N(j)} b_n \qquad \forall j \in J_{GPR} \tag{A.11}$$

$$(b_n - 1) \geq \sum_{g \in G(n)} (y_g - 1) \qquad \forall n \in N \tag{A.12}$$

$$b_n \leq y_g \qquad \forall n \in N, g \in G(n) \tag{A.13}$$

$$\sum_g (1 - y_g) \leq k \tag{A.14}$$

$$h_j^{lb} \leq h_j \leq h_j^{ub} \qquad \forall j \in J \tag{A.15}$$

$$d_j, b_n, y_g \in \{0,1\} \tag{A.16}$$

B. SimOptStrain

$$\max \quad \sum_j c_j v_j - \alpha \sum_g (1 - y_g) - \beta \sum_l z_l \tag{B.1}$$

$$\text{s.t.} \quad \sum_j S_{ij} v_j + \sum_l T_{il} w_l = 0 \qquad \forall i \in I : (u_i) \tag{B.2}$$

$$v_j \geq v_j^{lb} \qquad if\ d_j = 1 \qquad \forall j \in J_{LB} : (\lambda_j) \tag{B.3}$$

$$v_j = 0 \qquad if\ d_j = 0 \qquad \forall j \in J : (h_j) \tag{B.4}$$

$$w_l \geq w_l^{lb} \quad \text{if } z_l = 1 \quad \forall l \in L_{LB} : (\eta_l) \tag{B.5}$$

$$w_l = 0 \quad \text{if } z_l = 0 \quad \forall l \in L : (g_l) \tag{B.6}$$

$$\sum_i S_{ji} u_i = p_j \quad \text{if } d_j = 1 \quad \forall j \in J \setminus J_{LB} \tag{B.7}$$

$$\sum_i S_{ji} u_i - \lambda_j = p_j \quad \text{if } d_j = 1 \quad \forall j \in J_{LB} \tag{B.8}$$

$$\sum_i S_{ji} u_i + h_j = p_j \quad \text{if } d_j = 0 \quad \forall j \in J \tag{B.9}$$

$$\sum_i T_{li} u_i = 0 \quad \text{if } z_l = 1 \quad \forall l \in L \setminus L_{LB} \tag{B.10}$$

$$\sum_i T_{li} u_i - \eta_l = 0 \quad \text{if } z_l = 1 \quad \forall l \in L_{LB} \tag{B.11}$$

$$\sum_i T_{li} u_i + g_l = 0 \quad \text{if } z_l = 0 \quad \forall l \in L \tag{B.12}$$

$$\lambda_j \geq 0 \quad \forall j \in J_{LB} \tag{B.13}$$

$$\eta_l \geq 0 \quad \forall l \in L_{LB} \tag{B.14}$$

$$\sum_j p_j v_j = \sum_{j \in J_{LB}} (-v_j^{lb}) \lambda_j + \sum_{l \in L_{LB}} (-w_l^{lb}) \eta_l \tag{B.15}$$

$$d_j \geq b_n \quad \forall j \in J_{GPR}, n \in N(j) \tag{B.16}$$

$$d_j \leq \sum_{n \in N(j)} b_n \quad \forall j \in J_{GPR} \tag{B.17}$$

$$(b_n - 1) \geq \sum_{g \in G(n)} (y_g - 1) \quad \forall n \in N \tag{B.18}$$

$$b_n \leq y_g \quad \forall n \in N, g \in G(n) \tag{B.19}$$

$$\sum_g (1 - y_g) \leq k \tag{B.20}$$

$$\sum_l z_l \leq k' \tag{B.21}$$

$$h_j^{lb} \le h_j \le h_j^{ub} \qquad \forall j \in J \qquad \text{(B.22)}$$

$$d_j, b_n, y_g, z_l \in \{0,1\} \qquad \text{(B.23)}$$

C. BiMOMA

$$\max \quad \sum_j c_j v_j - \alpha \sum_g (1 - y_g) - \gamma \sum_j h_j^2 \qquad \text{(C.1)}$$

$$\text{s.t.} \quad \sum_j S_{ij} v_j = 0 \qquad \forall i \in I : (u_i) \qquad \text{(C.2)}$$

$$v_j \ge v_j^{lb} \qquad if\ \ d_j = 1 \qquad \forall j \in J_{LB} : (\lambda_j) \qquad \text{(C.3)}$$

$$v_j = 0 \qquad if\ \ d_j = 0 \qquad \forall j \in J : (h_j) \qquad \text{(C.4)}$$

$$\sum_i S_{ji} u_i = v_j - v_j^{wt} \qquad if\ \ d_j = 1 \qquad \forall j \in J \setminus J_{LB} \qquad \text{(C.5)}$$

$$\sum_i S_{ji} u_i + \lambda_j = v_j - v_j^{wt} \qquad if\ \ d_j = 1 \qquad \forall j \in J_{LB} \qquad \text{(C.6)}$$

$$\sum_i S_{ji} u_i + h_j = v_j - v_j^{wt} \qquad if\ \ d_j = 0 \qquad \forall j \in J \qquad \text{(C.7)}$$

$$\lambda_j \ge 0 \qquad \forall j \in J_{LB} \qquad \text{(C.8)}$$

$$\sum_j \frac{1}{2} v_j^2 - v_j^{wt} v_j \le \sum_j -\frac{1}{2} v_j^2 + \sum_{j \in J_{LB}} v_j^{lb} \lambda_j \qquad \text{(C.9)}$$

$$d_j \ge b_n \qquad \forall j \in J_{GPR}, n \in N(j) \qquad \text{(C.10)}$$

$$d_j \le \sum_{n \in N(j)} b_n \qquad \forall j \in J_{GPR} \qquad \text{(C.11)}$$

$$(b_n - 1) \ge \sum_{g \in G(n)} (y_g - 1) \qquad \forall n \in N \qquad \text{(C.12)}$$

$$b_n \le y_g \qquad \forall n \in N, g \in G(n) \qquad \text{(C.13)}$$

$$\sum_g (1 - y_g) \le k \qquad \text{(C.14)}$$

$$h_j^{lb} \leq h_j \leq h_j^{ub} \qquad \forall j \in J \qquad (C.15)$$

$$d_j, b_n, y_g \in \{0,1\} \qquad (C.16)$$
